# A diagnostic prediction model was established based on the clinical characteristics of multicenter children with Kawasaki disease in Xinjiang

**DOI:** 10.3389/fcvm.2025.1608572

**Published:** 2026-01-06

**Authors:** Muqing Niu, Zhaotang Lin, Fengling Zhang, Yonglin Chen, Li Zhang, Jinyong Pan, Cailing Chen, Hongbin Yang, Lian Mao, Lijuan Chen, Hua Guan, Yong Sun, Zhou Zhang, Jing Lv

**Affiliations:** 1 The First Affiliated Hospital of Shihezi University, Shihezi, Xinjiang, China; 2 Department of Pediatrics, The First Affiliated Hosptial of Shihezi University, Shihezi, Xinjiang, China; 3 Department of Pediatrics, First Division Hospital of Xinjiang Construction Corps, Alar, Xinjiang, China; 4 Department of Pediatrics, Second Division Hospital of Xinjiang Construction Corps, Tiemenguan, Xinjiang, China; 5 Department of Pediatrics, Third Division Hospital of Xinjiang Construction Corps, Tumushuke, Xinjiang, China; 6 Department of Pediatrics, Tumushuke People's Hospital, Tumushuke, Xinjiang, China; 7 Department of Pediatrics, Fourth Division Hospital of Xinjiang Construction Corps, Kekedala, Xinjiang, China; 8 Department of Pediatrics, Seventh Division Hospital of Xinjiang Construction Corps, Huyanghe, Xinjiang, China; 9 Department of Pediatrics, 10th Division Hospital of Xinjiang Construction Corps, Beitun, Xinjiang, China; 10 Department of Pediatrics, Red Star Hospital, 13th Division, Hami, Xinjiang, China

**Keywords:** CD4+ cells, CD8+ cells, coronary artery lesions, diagnostic biomarkers, immunoglobulins, intravenous immunoglobulin (IVIG), Kawasaki disease, lymphocyte subsets

## Abstract

**Introduction:**

Kawasaki disease (KD) is a serious pediatric systemic vasculitis that may cause cardiovascular complications, including coronary artery lesions (CAL). This study aimed to evaluate the predictive value of peripheral blood lymphocyte subsets for intravenous immunoglobulin (IVIG) treatment sensitivity in children with KD in Xinjiang.

**Methods:**

This multicenter retrospective study collected clinical and laboratory data from 142 children with KD and 120 controls with infectious fever across several hospitals in Xinjiang from June 2022 to December 2024. Peripheral blood lymphocyte subsets and immunoglobulins were analyzed. Multivariate logistic regression was used to identify risk factors for IVIG non-responsiveness, and ROC curve analyses were performed to evaluate diagnostic efficacy.

**Results:**

Significant differences in lymphocyte subsets were observed between KD and control groups, including CD3+, CD4+, CD8+, CD19+, and the CD4+/CD8+ ratio. Multivariate logistic regression identified CD4+ cell count as an independent risk factor for IVIG non-responsiveness. ROC analyses suggested that lymphocyte subsets and immunoglobulins (including CD3+, CD+, CD16+CD56+, IgA, and IgM) have diagnostic potential, with CD8+ and CD+ showing high sensitivity and specificity.

**Discussion:**

Peripheral blood lymphocyte subsets, particularly CD4+, may serve as useful biomarkers for predicting IVIG treatment response and distinguishing KD from other febrile illnesses. Further studies with larger sample sizes are warranted to refine predictive models and improve KD management.

## 1 Introduction

Kawasaki disease (KD) is an acute febrile exanthematous illness characterized by systemic vasculitis, predominantly affecting children. It may lead to coronary artery lesions (CAL), significantly compromising pediatric cardiovascular health. Intravenous immunoglobulin (IVIG) combined with aspirin remains the primary treatment during the acute phase. However, approximately 10%–15% of patients exhibit IVIG resistance (IVIG-nonresponsive KD), which is associated with a higher risk of CAL development (1). Early and accurate prediction of IVIG responsiveness and timely intervention are critical for improving clinical outcomes in children with KD (2–4).

Xinjiang, a region with unique geographical conditions, ethnic diversity, and lifestyle patterns, may present distinct pediatric disease profiles and epidemiological characteristics. Lymphocyte subsets play pivotal roles in immune regulation, and their alterations are closely linked to the pathogenesis of various diseases. Nevertheless, multicenter studies investigating the relationship between KD and lymphocyte subsets in Xinjiang children remain scarce. This multicenter retrospective study, conducted in collaboration with pediatric departments from multiple hospitals (including the First Division Hospital, Second Division Hospital, Third Division Hospital, Fourth Division Hospital, Fifth Division Hospital, Seventh Division Hospital, Tenth Division Hospital, and Hami Thirteenth Division Hongxing Hospital), aims to evaluate the predictive value of peripheral blood lymphocyte subset dynamics for IVIG treatment sensitivity in children with KD in Xinjiang (5). The findings may provide evidence-based insights for precision medicine in regional KD management.

## 2 Materials and methods

### 2.1 General information

This study adopts a multi-center retrospective design. Clinical case data were collected from 142 Kawasaki Disease (KD) hospitalized children diagnosed according to the KD diagnostic criteria at the following hospitals from June 2022 to December 2024: the Department of Pediatrics at Shihezi University First Affiliated Hospital, the Department of Pediatrics at the 1st Division Hospital of the Xinjiang Production and Construction Corps, the Department of Pediatrics at the Tumushuke People's Hospital, the Department of Pediatrics at the 5th Division Hospital of the Xinjiang Production and Construction Corps, and the Department of Pediatrics at the Bozhou City People's Hospital. In addition, 120 cases of children with infectious fever (mainly respiratory diseases such as acute suppurative tonsillitis, acute upper respiratory tract infection, acute bronchitis, bronchopneumonia, lobar pneumonia) hospitalized during the same period were included as the control group. The clinical data included general information, clinical manifestations, laboratory indicators, etc. Children who had a previous diagnosis of KD or autoimmune diseases, or those who had undergone immunosuppressive or corticosteroid therapy were excluded from the study (6).

General information, including ethnicity, age, sex, onset time, and common laboratory test indicators before and after intravenous IVIG treatment, were collected from the electronic medical record system of each hospital. Specialized personnel at each hospital were assigned to ensure the accuracy and completeness of the data, which was organized according to a standardized data collection form.

### 2.2 Inclusion criteria

KD Group Inclusion Criteria: Diagnosis of complete or incomplete KD based on the 2023 “Chinese Pediatric Kawasaki Disease Diagnosis and Treatment Guidelines”; Admission within the first 10 days of the acute phase of illness, aged 1–6 years, and the first treatment with IVIG (2 g/kg) combined with aspirin;Complete clinical data and peripheral blood lymphocyte subset testing before treatment (1).

Control Group Inclusion Criteria: 120 children aged 6 months to 5 years who were hospitalized with infectious fever (mainly respiratory system diseases such as acute suppurative tonsillitis, acute upper respiratory tract infection, acute bronchitis, bronchopneumonia, lobar pneumonia) during the same period.

### 2.3 Exclusion criteria

Exclusion criteria include: 1) autoimmune diseases, such as systemic lupus erythematosus, rheumatic fever, rheumatoid arthritis, dermatomyositis, etc.; 2) immunodeficiency diseases, including congenital and acquired immune deficiencies; 3) hematological diseases or malignancies, such as leukemia, aplastic anemia, primary thrombocytopenia; 4) endocrine diseases, such as growth hormone deficiency, precocious puberty, hyperthyroidism, hypothyroidism; 5) congenital heart diseases, such as patent ductus arteriosus, ventricular septal defect, tetralogy of Fallot, pulmonary valve stenosis, and complex structural heart diseases; 6) genetic metabolic diseases, such as phenylketonuria, trisomy 21 syndrome, etc.; 7) prior use of IVIG or corticosteroid therapy during the diagnostic or therapeutic process; 8) allergic reactions to the drugs used in the study; 9) Incomplete clinical case or laboratory test data (1, 4–7).

### 2.4 Lymphocyte subset detection

Retrospective collection of 2 mL fasting venous blood from all enrolled subjects before treatment, placed in heparin anti-coagulant tubes. The storage conditions were recorded based on the actual circumstances of each hospital (some hospitals may store samples at −80 °C). BD Multitest 6-color TBNK reagent (BD Biosciences, USA) and absolute counting microsphere kits (Beijing Tongsheng Shidai, China) were used to detect lymphocyte subsets by flow cytometry (Beckman Coulter, USA). The tested indicators included the total T cells (CD3+), CD4+ T cells (CD3 + CD4+), CD8+ T cells (CD3 + CD8+), B cells (CD3-CD19+), and natural killer (NK) cells (CD16+/CD56+) ratios and absolute count levels. The testing method followed the standard operating procedures for flow cytometry provided by Cell Signaling Technology (CST) (8, 9).

To ensure consistency and accuracy of the results, each hospital performed instrument calibration, personnel training before the tests, and regular inter-laboratory quality assessments.

### 2.5 Statistical analysis

The data were analyzed using R 4.4.2 software. Continuous data were presented as mean ± standard deviation (*x* ± *s*), and categorical data were presented as percentages (%). For comparisons between multiple groups, one-way analysis of variance (ANOVA) was used, and pairwise comparisons were performed using the LSD test. Chi-square (*χ*^2^) test was used for comparisons of categorical data. Variables with statistical significance in univariate analysis were included in multivariate logistic regression analysis to identify risk factors influencing IVIG treatment sensitivity (10). A *P*-value of <0.05 was considered statistically significant. After data collection, the statistical analysis was conducted by the School of Medicine, Shihezi University, ensuring the standardization and scientific nature of the analysis process.

### 2.6 Definition of IVIG responsiveness

**Figure 1:**
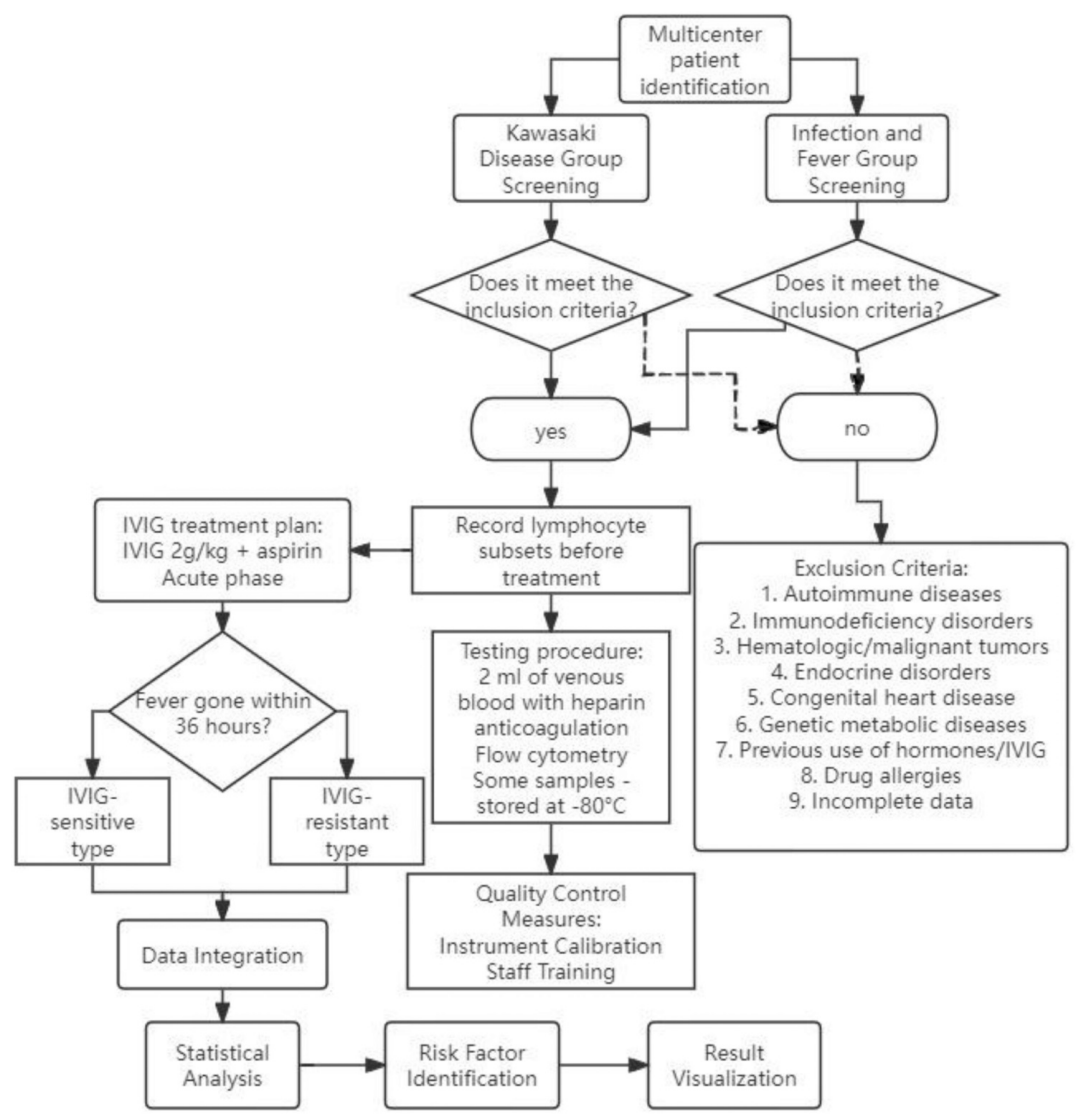
Detailed study workflow diagram. It illustrates patient enrollment, inclusion/exclusion criteria, grouping, laboratory testing, and statistical analysis steps.

IVIG responsiveness was defined as the resolution of fever within 36 h following completion of the initial IVIG infusion (2 g/kg). IVIG non-responsiveness (resistance) was defined as persistent or recrudescent fever (≥38.0 °C) occurring between 36 h and 7 days after the initial IVIG administration, in accordance with the 2023 Chinese Pediatric Kawasaki Disease Guidelines (1). All processes are shown in Figure 1.

## 3 Results

### 3.1 Clinical data between the Two groups

A total of 142 children were included in the KD group, with 83 males (58.6%) and 59 females (41.4%), with a mean age of (3.91 ± 1.12) years. The infectious fever group included 120 children, with 63 males (52.3%) and 57 females (47.7%), with a mean age of (3.99 ± 1.11) years. There were no significant differences in age or gender between the two groups.

### 3.2 Comparison of laboratory results between the two groups

Laboratory test results including white blood cell (WBC) count, red blood cell (RBC) count, monocyte count (MO), neutrophil count (NE), lymphocyte count (LY), hemoglobin (HB), C-reactive protein (CRP), erythrocyte sedimentation rate (ESR), alanine aminotransferase (ALT), aspartate aminotransferase (AST), and creatine kinase-MB (CK-MB) were collected and analyzed. Key Findings: WBC, CRP, NE, PLT, ALT: The levels in the KD group were significantly higher than those in the infectious fever group, with *P* < 0.001 (11). ALB, HB: The levels in the KD group were significantly lower than those in the infectious fever group, with *P* < 0.001. There were no significant differences between the two groups in terms of LY, MO, ESR, LDH, Na+, AST, and CK-MB (*P* > 0.05). Conclusion: Significant differences were observed in the levels of WBC, CRP, NE, PLT, ALT, ALB, and HB between the two groups (*P* < 0.05). No significant differences were found for LY, MO, ESR, LDH, Na+, AST, and CK-MB between the two groups (*P* > 0.05) (Table 1).

**Table 1:** Baseline demographic and laboratory characteristics of children with Kawasaki disease and infectious fever.

Variables	KD group (*n* = 142)	Infectious fever group (*n* = 120)	*Z*/*t*/*χ*²	*P* value
Sex, male/female, *n* (%)	83/59 (58.6/41.4)	63/57 (52.3/47.7)	0.708	0.400
Age (years), mean ± SD	3.91 ± 1.12	3.99 ± 1.11	1.334	0.249
WBC (×10^9^/L), M (P25, P75)	14.73 (12.91, 16.26)	9.84 (8.86, 10.82)	20.43	<0.001
CRP (mg/L), M (P25, P75)	44.83 (26.82, 58.20)	12.51 (11.26, 13.76)	15.66	<0.001
NE (×10^9^/L), M (P25, P75)	9.48 (7.09, 11.54)	4.78 (4.30, 5.26)	15.94	<0.001
LY (×10^9^/L), M (P25, P75)	3.51 (2.72, 4.41)	3.10 (2.79, 3.41)	−0.249	0.803
MO (×10^9^/L), M (P25, P75)	1.01 (0.81, 1.26)	0.88 (0.79, 0.97)	1.17	0.242
ESR (mm/h), M (P25, P75)	63.74 (60.81, 66.61)	64.04 (57.64, 70.44)	−0.52	0.600
PLT (×10^9^/L), M (P25, P75)	386.82 (322.72, 446.54)	260.06 (234.05, 286.07)	16.32	<0.001
HB (g/L), M (P25, P75)	111.50 (106.90, 116.49)	120.80 (108.72, 132.88)	−6.65	<0.001
AST (U/L), M (P25, P75)	46.91 (43.81, 50.04)	47.32 (42.59, 52.05)	−0.60	0.551
ALT (U/L), M (P25, P75)	66.57 (43.71, 88.95)	51.95 (34.64, 69.26)	5.35	<0.001
CK (U/L), M (P25, P75)	65.87 (44.57, 82.13)	68.19 (61.37, 75.01)	−1.46	0.145
CK-MB (U/L), M (P25, P75)	34.01 (26.47, 40.74)	32.47 (29.22, 35.72)	1.13	0.260
LDH (U/L), M (P25, P75)	338.93 (307.43, 377.84)	331.21 (298.09, 364.33)	1.60	0.109
ALB (g/L), mean ± SD	34.05 ± 6.72	37.56 ± 4.39	−4.90	<0.001
Na^+^ (mmol/L), M (P25, P75)	136.25 (134.92, 137.37)	137.10 (123.39, 150.81)	−0.70	0.484

### 3.3 Comparison of lymphocyte subsets between the two groups

Compared to the infectious fever group, the acute phase KD group showed the following differences in lymphocyte subset counts: CD4+ cells were significantly lower in the KD group (*P* < 0.05) (6–8, 12). CD3+ cells, CD8+ cells, and CD16 + CD56+ cells absolute counts were significantly lower in the KD group (*P* < 0.001). CD19+ cells and the CD4+/CD8+ ratio were significantly higher in the KD group (*P* < 0.001).

These findings suggest that there are notable changes in the lymphocyte subset distribution in KD patients during the acute phase when compared to children with infectious fever (Table 2).

**Table 2:** Comparison of lymphocyte subsets between the KD group and the infectious fever group.

Laboratory test	KD group (Mean ± SD)	Infectious fever group (Mean ± SD)	Z/t	*P*
CD3 (cells/*μ*L)	2,018.20 ± 501.05	2,675.41 ± 546.45	−10.15	<0.001
CD4 (cells/μL)	1,265.49 ± 368.68	1,356.69 ± 329.13	−2.09	0.037
CD8 (cells/μL)	667.16 ± 198.98	1,044.14 ± 227.43	−14.31	<0.001
CD4/CD8 Ratio	2.0 ± 0.70	1.36 ± 0.50	8.37	<0.001
CD16CD56 (cells/μL)	314.99 ± 106.55	420.38 ± 108.28	−7.92	<0.001
CD19 (cells/μL)	1,164.69 ± 257.13	1,050.73 ± 87.26	4.63	<0.001

### 3.4 Comparison of immunoglobulin levels between the Two groups

There were statistically significant differences in the levels of IgA, IgG, and IgM between the KD group and the infectious fever group (*P* < 0.05). Specifically: IgA and IgG levels were significantly higher in the infectious fever group compared to the KD group (10, 11, 13). IgM and C3 levels were significantly higher in the KD group compared to the infectious fever group. There was no significant difference in C4 levels between the two groups (*P* > 0.05).

These findings indicate distinct immunoglobulin responses between the two groups, with differences in specific immunoglobulins reflecting different immune responses in KD and infectious fever (Supplementary Table S1).

### 3.5 Multivariate logistic regression analysis of lymphocyte subsets and immunoglobulins

Using the diagnosis of Kawasaki disease as the dependent variable, the variables with significant differences from univariate analysis, including IgA, IgM, IgG, absolute counts of CD3, CD4, CD8, CD16CD56 cells, and the CD4/CD8 ratio, were selected as independent variables. A multivariate logistic regression analysis was performed to evaluate the relationship between the selected independent variables and the diagnosis of Kawasaki disease (Supplementary Table S2).

### 3.6 Analysis of lymphocyte subsets and immunoglobulins for diagnosing Kawasaki disease

Using the diagnosis of Kawasaki disease as the dependent variable, we selected the variables with significant differences in lymphocyte subsets and immunoglobulins. The diagnostic efficacy of CD3+, CD8+, CD16 + CD56+, IgA, and IgM was evaluated using ROC curves: AUC values for the ROC curves for CD3+, CD8+, CD16 + CD56+, IgA, and IgM in diagnosing Kawasaki disease were 0.816, 0.887, 0.765, 0.723, and 0.715, respectively, all of which are greater than 0.5, indicating good diagnostic potential. The sensitivity and specificity for each marker were as follows: CD3+: Sensitivity = 84.2%, Specificity = 67.6%; CD8+: Sensitivity = 85.0%, Specificity = 80.3%; CD16 + CD56+: Sensitivity = 81.7%, Specificity = 63.4%; IgA: Sensitivity = 61.7%, Specificity = 83.8%;IgM: Sensitivity = 61.8%, Specificity = 74.6%.

These findings suggest that CD3+, CD8+, CD16 + CD56+, IgA, and IgM are reliable biomarkers for diagnosing Kawasaki disease, with CD8 + and CD3 + showing the highest sensitivity and specificity (Table 3).

**Table 3:** Diagnostic efficacy of lymphocyte subsets and immunoglobulins for Kawasaki disease.

Lymphocyte subset/immunoglobulin	AUC	95% confidence interval	Sensitivity	Specificity	Optimal cutoff value
CD3	0.816	0.767–0.865	84.20%	67.60%	2,198.28
CD8	0.887	0.846–0.925	85.00%	80.30%	827.67
CD16CD56	0.765	0.706–0.824	81.70%	63.40%	336.27
IgA	0.723	0.655–0.787	61.70%	83.80%	1.25
IgM	0.715	0.650–0.777	61.80%	74.60%	7.8

### 3.7 Comparison of laboratory indicators between IVIG responders and non-responders

Compared with IVIG responders, IVIG non-responders exhibited significantly higher WBC, CRP, NE, PLT, and ALT levels and lower HB and ALB levels (*P* < 0.05). No significant differences were observed in LY, MO, ESR, LDH, Na^+^, AST, CK-MB, or C3/C4 levels (Supplementary Table S3).

### 3.8 Comparison of lymphocyte subsets between IVIG responders and non-responders

The IVIG non-responder group demonstrated significantly lower CD4^+^ T cell counts than the responder group (*P* < 0.05). No significant differences were found in CD3^+^, CD8^+^, CD4/CD8 ratio, CD16^+^CD56^+^, or CD19^+^ counts (*P* > 0.05).

### 3.9 Comparison of immunoglobulins and complement levels between IVIG responders and non-responders

There were no statistically significant differences between the IVIG sensitive group and the IVIG non-responders group for IgM, IgA, IgG, C3, and C4 levels (*P* > 0.05).

This suggests that immunoglobulin and complement levels do not differ significantly between IVIG responders and non-responders, implying that these biomarkers may not be effective in distinguishing between the two groups in the context of Kawasaki disease (Table 4).

**Table 4:** Comparison of immunoglobulins and complement levels between IVIG sensitive and non-responders groups.

Indicator	IVIG responders (*n* = 108)	IVIG non-responders (*n* = 34)	*t*/*Z*	*P* value
CD3^+^ (cells/μL)	2,042.22 ± 518.74	1,941.91 ± 438.51	1.02	0.31
CD4^+^ (cells/μL)	1,304.68 ± 387.90	1,140.99 ± 268.16	2.29	0.02
CD8^+^ (cells/μL)	667.17 ± 198.74	667.11 ± 202.75	0.00	0.99
CD4/CD8 ratio	2.03 ± 0.69	1.91 ± 0.72	0.88	0.38
CD16^+^CD56^+^ (cells/μL)	315.90 ± 110.91	312.08 ± 92.78	0.18	0.86
CD19^+^ (cells/μL)	1,181.10 ± 270.11	1,112.58 ± 205.55	1.36	0.18
IgM (g/L)	1.13 ± 0.32	1.12 ± 0.43	0.116	0.908
IgA (g/L)	0.89 ± 0.43	0.96 ± 0.40	−0.872	0.387
IgG (g/L)	6.65 ± 1.84	6.70 ± 1.64	—	>0.05
C3 (g/L)	1.32 ± 0.20	1.34 ± 0.23	—	>0.05
C4 (g/L)	0.29 ± 0.10	0.29 ± 0.09	—	>0.05

### 3.10 Univariate logistic regression analysis of factors affecting IVIG sensitivity

In the univariate analysis, CD4+ cell count was selected as the independent variable based on its significant difference in the previous analysis. The results of the univariate logistic regression analysis showed that a decrease in CD4+ cell count is an independent risk factor for IVIG non-responsiveness (*P* < 0.05).

This suggests that lower CD4+ cell counts are associated with a reduced likelihood of responding to IVIG treatment in Kawasaki disease patients (Supplementary Table S4).

### 3.11 ROC curve analysis for predicting IVIG non-responsiveness and coronary artery dilatation in Kawasaki disease

**Figure 2:**
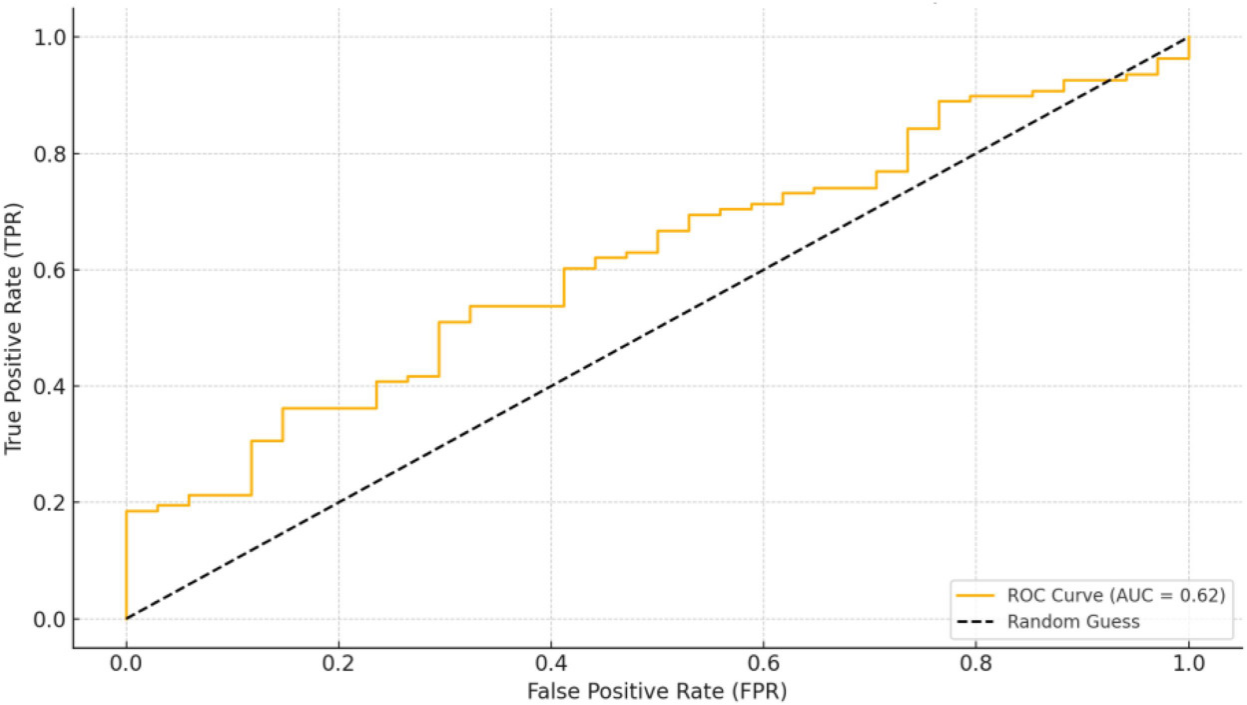
ROC graph.

The ROC curves for IgG, CD8, and their combination were plotted to assess their predictive value for the occurrence of coronary artery dilatation in Kawasaki disease patients (as shown in Figure 2). The analysis calculated the Area Under the Curve (AUC), Youden Index, Sensitivity, and Specificity, and selected the optimal cutoff values (14) (Supplementary Table S5).

## 4 Discussion

In this multicenter retrospective study from Xinjiang, we comprehensively compared routine inflammatory indices, lymphocyte subsets, immunoglobulins and complement between children with KD and those with infectious fever, and further explored the association between these immune markers and IVIG responsiveness (15). Three main findings emerged: (1) KD was characterized by more intense systemic inflammation and a distinct pattern of lymphocyte subset and immunoglobulin alterations compared with infectious fever; (2) CD3^+^, CD8^+^, CD16^+^CD56^+^ cells, IgA and IgM showed good diagnostic performance for differentiating KD from infectious fever, with CD8^+^ and CD3^+^ cells providing the highest AUC values; and (3) a lower CD4^+^ T-cell count was identified as an independent risk factor for IVIG non-responsiveness (16).

Compared with the infectious fever group, children with KD had significantly higher WBC, CRP, NE, PLT and ALT levels, but lower ALB and HB levels, reflecting the profound systemic inflammatory response, hepatocellular injury and capillary leakage that characterize the acute phase of KD. These findings are broadly consistent with previous reports and with traditional risk scores for IVIG resistance that are based on routine laboratory parameters combined with clinical features. However, because such markers are not specific for KD and are also elevated in many other febrile illnesses, there is a clear need for additional biomarkers that can better distinguish KD from infection and refine risk stratification.

More pronounced differences between KD and infectious fever were observed in peripheral lymphocyte subsets. KD patients showed reduced absolute counts of CD3^+^, CD4^+^, CD8^+^ and CD16^+^CD56^+^ cells, together with higher CD19^+^ counts and an increased CD4^+^/CD8^+^ ratio. The relatively greater decline in CD8^+^ than in CD4^+^ cells, resulting in a higher CD4^+^/CD8^+^ ratio, suggests an imbalance between helper and cytotoxic T-cell populations and may contribute to sustained immune activation and vascular inflammation. At the same time, the increase in CD19^+^ B cells and the distinct immunoglobulin profile—lower IgA and IgG but higher IgM in KD—indicate dynamic changes in humoral immunity and different patterns of antigen exposure or class switching compared with infectious fever.

From a diagnostic standpoint, our ROC analysis showed that CD3^+^, CD8^+^ and CD16^+^CD56^+^ cells, together with IgA and IgM, had meaningful discriminatory value for identifying KD among febrile children. CD8^+^ T cells, in particular, achieved the best diagnostic performance, followed by CD3^+^ T cells. These results suggest that quantitative assessment of lymphocyte subsets may complement conventional inflammatory markers and clinical features in the early identification of KD. Given that flow-cytometric lymphocyte subset analysis is increasingly available in tertiary pediatric centers and can be performed on small blood samples, integrating these parameters into diagnostic algorithms for children with persistent fever may be feasible in routine practice.

A novel and clinically relevant finding of our study is that a lower CD4^+^ T-cell count was independently associated with IVIG non-responsiveness. CD4^+^ T cells play a central role in coordinating both cellular and humoral immune responses, including the regulation of pro-inflammatory cytokines and the provision of help to B cells. Quantitative depletion of CD4^+^ cells may therefore reflect profound immune dysregulation and an impaired capacity to mount an appropriate regulatory response to IVIG, predisposing patients to persistent inflammation and a higher risk of coronary artery involvement. Our results indicate that KD patients with low baseline CD4^+^ T-cell counts may warrant closer echocardiographic follow-up and consideration of more intensive initial therapy or early escalation when fever persists after IVIG.

This study has several strengths, including its multicenter design in a region with distinctive ethnic and environmental characteristics, and the simultaneous evaluation of immune markers for both diagnosis and prediction of IVIG responsiveness using an infectious fever control group. Nonetheless, several limitations should be noted. The retrospective design may introduce selection bias, and the sample size—particularly for IVIG non-responders and patients with coronary artery dilatation—remains modest. We did not include healthy controls, and more detailed immunological profiling (such as T-helper subsets, regulatory T cells, cytokines or genetic markers) was not available. In addition, our models were derived from a single regional cohort and require external validation in other populations.

In summary, our findings highlight the important role of lymphocyte subsets and immunoglobulins in both the diagnosis of KD and the prediction of IVIG responsiveness in children from Xinjiang. Decreased CD4^+^ T-cell counts may serve as a simple, clinically accessible indicator of IVIG non-responsiveness, whereas CD8^+^ and CD3^+^ T cells offer useful discriminatory power for differentiating KD from infectious fever. Future prospective, multicenter studies with more comprehensive immune profiling are warranted to validate and refine immune-based diagnostic and prognostic tools for KD.

## 5 Conclusion

This study highlights the significant role of immune markers, particularly lymphocyte subsets, in the diagnosis and treatment response of Kawasaki disease (KD) in children from Xinjiang. The findings demonstrate that CD4+ cell counts can serve as an important marker for predicting IVIG sensitivity, with a decrease in CD4+ cells being an independent risk factor for IVIG non-responsiveness. The study also shows notable differences in lymphocyte subsets between KD patients and children with infectious fever, which may aid in distinguishing KD from other febrile illnesses. Furthermore, the research provides valuable insights into the immune dysregulation associated with KD, particularly T cell activation and immune imbalance, which contribute to the vascular inflammation seen in the disease. These findings could help improve early diagnosis and treatment strategies for KD, although further studies with larger sample sizes and additional immune markers are needed to refine the diagnostic and predictive models (6, 8, 9, 14, 17).

## Data Availability

The raw data supporting the conclusions of this article will be made available by the authors, without undue reservation.

## References

[1] Center for Diagnosis and Treatment of Kawasaki Disease/Children’s Hospital of Shaanxi Provincial People’s Hospital, National Children’s Medical Center/Beijing Children’s Hospital, Capital Medical University, Children’s Hospital, Shanghai Jiao Tong University School of Medicine, National Regional Medical Center/Shengjing Hospital of China Medical University Evidence-based guidelines for the diagnosis and treatment of Kawasaki disease in children in China (2023). Zhongguo Dang Dai Er Ke Za Zhi. (2023) 25(12):1198–210.38112136

[2] Newburger JW Takahashi M Burns JC Beiser AS Chung KJ Duffy CE The treatment of Kawasaki syndrome with intravenous gamma globulin. N Engl J Med. (1986) 315(6):341–7. 10.1056/NEJM198608073150601 2426590

[3] Subspecialty Group of Cardiology, the Society of Pediatrics, Chinese Medical Association, Subspecialty Group of Rheumatology, the Society of Pediatrics, Chinese Medical Association The expert consensus on diagnosis and acute-phase treatment of Kawasaki disease. Zhonghua Er Ke Za Zhi. (2022) 60(1):6–13. 34986616 10.3760/cma.j.cn112140-20211018-00879

[4] Kawasaki T. Acute febrile mucocutaneous syndrome with lymphoid involvement with specific desquamation of the fingers and toes in children. Arerugi. (1967) 16(3):178–222. 6062087

[5] Ae R Abrams JY Maddox RA Schonberger LB Nakamura Y Kuwabara M Corticosteroids added to initial intravenous immunoglobulin treatment for the prevention of coronary artery abnormalities in high-risk patients with Kawasaki disease. J Am Heart Assoc. (2020) 9(17):e015308. 10.1161/JAHA.119.015308 32811256 PMC7660775

[6] Hamada H Suzuki H Onouchi Y Ebata R Terai M Fuse S Efficacy of primary treatment with immunoglobulin plus ciclosporin for prevention of coronary artery abnormalities in patients with Kawasaki disease predicted to be at increased risk of non-response to intravenous immunoglobulin (KAICA): a randomised controlled, open-label, blinded-endpoints, phase 3 trial. Lancet. (2019) 393(10176):1128–37. 10.1016/S0140-6736(18)32003-8 30853151

[7] Wang Y Cao Y Li Y Zhu F Yuan M Xu J Development of an immunoinflammatory indicator-related dynamic nomogram based on machine learning for the prediction of intravenous immunoglobulin-resistant Kawasaki disease patients. Int Immunopharmacol. (2024) 134:112194. 10.1016/j.intimp.2024.112194 38703570

[8] Noval Rivas M Lee Y Wakita D Chiba N Dagvadorj J Shimada K CD8+ T cells contribute to the development of coronary arteritis in the Lactobacillus casei cell wall extract-induced murine model of Kawasaki disease. Arthritis Rheumatol. (2017) 69(2):410–21. 10.1002/art.39939 27696768 PMC5274597

[9] Xie Z Huang Y Li X Lun Y Li X He Y Atlas of circulating immune cells in Kawasaki disease. Int Immunopharmacol. (2022) 102:108396. 10.1016/j.intimp.2021.108396 34890998

[10] Seo E Yu JJ Jun HO Shin EJ Baek JS Kim YH Prediction of unresponsiveness to second intravenous immunoglobulin treatment in patients with Kawasaki disease refractory to initial treatment. Korean J Pediatr. (2016) 59(10):408–13. 10.3345/kjp.2016.59.10.408 27826327 PMC5099288

[11] Yang S Song R Zhang J Li X Li C. Predictive tool for intravenous immunoglobulin resistance of Kawasaki disease in Beijing. Arch Dis Child. (2019) 104(3):262–7. 10.1136/archdischild-2017-314512 30026252

[12] Kuo HC Wang CL Liang CD Yu HR Huang CF Wang L Association of lower eosinophil-related T helper 2 (Th2) cytokines with coronary artery lesions in Kawasaki disease. Pediatr Allergy Immunol. (2009) 20(3):266–72. 10.1111/j.1399-3038.2008.00779.x 19438983

[13] Lu Y Chen T Wen Y Si F Wu X Yang Y. Prediction of repeated intravenous immunoglobulin resistance in children with Kawasaki disease. BMC Pediatr. (2021) 21(1):406. 10.1186/s12887-021-02876-w 34530763 PMC8444587

[14] Duan M Geng Z Gao L Zhao Y Li Z Chen L An interpretable machine learning-assisted diagnostic model for Kawasaki disease in children. Sci Rep. (2025) 15(1):7927. 10.1038/s41598-025-92277-1 40050685 PMC11885592

[15] McCrindle BW Rowley AH Newburger JW Burns JC Bolger AF Gewitz M Diagnosis, treatment, and long-term management of Kawasaki disease: a scientific statement for health professionals from the American Heart Association. Circulation. (2017) 135(17):e927–99. 10.1161/CIR.0000000000000484 28356445

[16] Matsuguma C Wakiguchi H Suzuki Y Okada S Furuta T Ohnishi Y Dynamics of immunocyte activation during intravenous immunoglobulin treatment in Kawasaki disease. Scand J Rheumatol. (2019) 48(6):491–6. 10.1080/03009742.2019.1604992 31272272

[17] Pan Y Jiao F. Exploring causal correlations between inflammatory cytokines and Kawasaki disease: a Mendelian randomization. Fetal Pediatr Pathol. (2024) 44:1–13. 10.1080/15513815.2024.2414175 39485066

