# Supplementary material for: A diagnostic prediction model was established based on the clinical characteristics of multicenter children with Kawasaki disease in Xinjiang
Source: Front Cardiovasc Med. 2026 Jan 6;12:1608572. doi: 10.3389/fcvm.2025.1608572 (PMC12816189; doi:10.3389/fcvm.2025.1608572)
Supplement: Supplementary file 1 [file Table1.docx]

Table 1 Comparison of Immunoglobulin and Complement Levels Between the KD Group and the Infectious Fever Group

| Group | n | IgM (g/L) | IgG (g/L) | IgA (g/L) | t | P |
| --- | --- | --- | --- | --- | --- | --- |
| KD Group | 142 | 1.12 ± 0.35 | 6.66 ± 1.79 | 0.91 ± 0.42 | 2.99 | 0.003 |
| Infectious Fever Group | 120 | 1.00 ± 0.30 | 8.26 ± 2.35 | 1.45 ± 0.76 | -6.124 | <0.001 |

Table 2 Multivariate Logistic Regression Analysis of Lymphocyte Subsets and Immunoglobulins

| Variable | Regression Coefficient | Standard Error | Wald | P | OR |
| --- | --- | --- | --- | --- | --- |
| CD3 (cells/μL) | -0.002 | 0 | 17.662 | 0 | 0.998 |
| CD4 (cells/μL) | 0 | 0.001 | 0.114 | 0.736 | 1 |
| CD8 (cells/μL) | -0.006 | 0.001 | 19.574 | 0 | 0.994 |
| CD4/CD8 | 0.88 | 0.515 | 2.925 | 0.087 | 2.411 |
| CD19 (cells/μL) | 0.002 | 0.001 | 1.34 | 0.247 | 1.002 |
| CD16CD56 (cells/μL) | -0.008 | 0.002 | 13.1 | 0 | 0.992 |
| IgM | 1.026 | 0.7 | 2.146 | 0.143 | 2.791 |
| IgA | -1.259 | 0.417 | 9.13 | 0.003 | 0.284 |
| IgG | -0.331 | 0.121 | 7.49 | 0.006 | 0.718 |

Comparison Between IVIG Non-responders and IVIG Responders

Table 3 Laboratory Tests Comparison Between IVIG Non-responders and IVIG Responders

| Laboratory Test | IVIG Non-responders Group (M (P25, P75)) | IVIG Responders Group (M (P25, P75)) | Z/t | P |
| --- | --- | --- | --- | --- |
| WBC (×10^9/L) | 14.73 (12.91, 16.26) | 9.84 (8.86, 10.82) | 20.43 | <0.001 |
| CRP (mg/L) | 44.83 (26.82, 58.20) | 12.51 (11.26, 13.76) | 15.66 | <0.001 |
| NE (×10^9/L) | 9.48 (7.09, 11.54) | 4.78 (4.30, 5.26) | 15.94 | <0.001 |
| LY (×10^9/L) | 3.51 (2.72, 4.41) | 3.10 (2.79, 3.41) | -0.249 | 0.803 |
| MO (×10^9/L) | 1.01 (0.81, 1.26) | 0.88 (0.79, 0.97) | 1.17 | 0.242 |
| ESR (mm/h) | 63.74 (60.81, 66.61) | 64.04 (57.64, 70.44) | -0.52 | 0.6 |
| PLT | 386.82 (322.72, 446.54) | 260.06 (234.05, 286.07) | 16.32 | <0.001 |
| HB (g/L) | 111.50 (106.90, 116.49) | 120.8 (108.72, 132.88) | -6.65 | <0.001 |
| AST (U/L) | 46.91 (43.81, 50.04) | 47.32 (42.59, 52.05) | -0.6 | 0.551 |
| ALT (U/L) | 66.57 (43.71, 88.95) | 51.95 (34.64, 69.26) | 5.35 | <0.001 |
| CK (U/L) | 65.87 (44.57, 82.13) | 68.19 (61.37, 75.01) | -1.46 | 0.145 |
| CK-MB (U/L) | 34.01 (26.47, 40.74) | 32.47 (29.22, 35.72) | 1.13 | 0.26 |
| LDH (U/L) | 338.93 (307.43, 377.84) | 331.21 (298.09, 364.33) | 1.6 | 0.109 |
| ALB (g/L) | 34.05 ± 6.72 | 37.56 ± 4.39 | -4.9 | <0.001 |
| Na+ | 136.25 (134.92, 137.37) | 137.10 (123.39, 150.81) | -0.7 | 0.484 |

Table 4 Logistic Regression Analysis of Factors Influencing IVIG Non-responsiveness in Kawasaki Disease

| Indicator | B | SE | Wald | P | OR | 95% CI (Lower Limit) | 95% CI (Upper Limit) |
| --- | --- | --- | --- | --- | --- | --- | --- |
| CD4 | -0.001 | 0.001 | 4.946 | 0.026 | 0.999 | 0.997 | 1 |

Table 5 Diagnostic Efficacy of CD4+ for IVIG Non-responders in Kawasaki Disease

| Lymphocyte Subset | AUC | 95% Confidence Interval | Sensitivity | Specificity | Youden Index | Optimal Cutoff Value |
| --- | --- | --- | --- | --- | --- | --- |
| CD4+ | 0.616 | 0.518 ~ 0.716 | 70.59% | 50.93% | 0.215 | 1261.61 |
